# Prevalence of Asymptomatic SARS-CoV-2 Infection in Japan

**DOI:** 10.1001/jamanetworkopen.2022.47704

**Published:** 2022-12-27

**Authors:** Toru Suzuki, Kenichi Aizawa, Kenji Shibuya, Shinya Yamanaka, Yuichiro Anzai, Kiyoshi Kurokawa, Ryozo Nagai

**Affiliations:** 1 Jichi Medical University, Tochigi, Japan; 2 The Institute of Medical Science, The University of Tokyo, Tokyo, Japan; 3 University of Leicester, NIHR Leicester Biomedical Research Centre, Leicester, United Kingdom; 4 Tokyo Foundation for Policy Research, Tokyo, Japan; 5 Center for iPS Cell Research and Application, Kyoto University, Kyoto, Japan; 6 The Advisory Board of the COVID-19 Artificial Intelligence Simulation Project promoted by the Office for COVID-19 and Other Emerging Infectious Disease Control, Cabinet Secretariat, Government of Japan; 7 Health and Global Policy Institute, Tokyo, Japan; 8 Division of Clinical Pharmacology, Department of Pharmacology, Jichi Medical University, Tochigi, Japan

## Abstract

**Importance:**

Real-world evidence of SARS-CoV-2 transmission is needed to understand the prevalence of infection in the Japanese population.

**Objective:**

To conduct sentinel screening of the Japanese population to determine the prevalence of SARS-CoV-2 infection in asymptomatic individuals, with complementary analysis for symptomatic patients as reported by active epidemiologic surveillance used by the government.

**Design, Setting, and Participants:**

This cross-sectional study of a sentinel screening program investigated approximately 1 million asymptomatic individuals with polymerase chain reaction (PCR) testing for SARS-CoV-2 infection between February 22 and December 8, 2021. Participants included children, students, employed adults, and older individuals, as well as volunteers to broadly reflect the general Japanese population in the 14 prefectures of Japan that declared a state of emergency. Saliva samples and a cycle threshold (Ct) value of approximately 40 as standard in Japan were used. Polymerase chain reaction testing for symptomatic patients was separately done by public health authorities, and the results were obtained from the Ministry of Health, Labour, and Welfare of Japan to complement data on asymptomatic infections from the present study.

**Main Outcomes and Measures:**

Temporal trends in positivity and prevalence (including surges of different variants) and demographic associations (eg, age, geographic location, and vaccination status) were assessed.

**Results:**

The positive rate of SARS-CoV-2 infection in 1 082 976 asymptomatic individuals (52.08% males; mean [SD] age 39.4 [15.7] years) was 0.03% (95% CI, 0.02%-0.05%) during periods without surges and a maximum of 0.33% (95% CI, 0.25%-0.43%) during peak surges at the Japanese standard Ct value of approximately 40; however, the positive rate would have been 10-fold less at a Ct value of 25 as used elsewhere in the world (eg, UK). There was an increase in patients with a positive PCR test result with a Ct value of 25 or 30 preceding surges in infection and hotspots of asymptomatic infections.

**Conclusions and Relevance:**

In this cross-sectional study of asymptomatic SARS-CoV-2 infection in the general population of Japan in 2021, as investigated by sentinel surveillance, a low rate of infection was seen in the Japanese population compared with reported levels elsewhere in the world. This finding provides real-world data on the state of infection in Japan.

## Key Points

**Question:**

Would investigation of asymptomatic SARS-CoV-2 add to the understanding of the state of infection in the community and population of Japan by complementing available data on symptomatic infections?

**Findings:**

In this cross-sectional study of 1 million individuals in Japan with asymptomatic SARS-CoV-2 infection, the prevalence of infection was generally lower than in similar studies from other nations. An inverse association between vaccination rate and age group for asymptomatic infections and symptomatic patients was seen, with younger age groups with lower vaccination rates including more symptomatic patients and asymptomatic infections.

**Meaning:**

The findings of this cross-sectional study suggest that Japan had a low rate of SARS-CoV-2 infection in the general population compared with other countries.

## Teaser

This cross-sectional study examines the prevalence of asymptomatic SARS-CoV-2 infection in Japan.

## Introduction

The Asia-Pacific region, including Japan, has had fewer reported COVID-19 cases compared with Europe and the Americas during the SARS-CoV-2 pandemic.^1^ Infections in Japan have been recorded based on positive results of standard polymerase chain reaction (PCR) tests within the framework of active epidemiologic surveillance managed by public health authorities.^2,3,4,5^ However, the number of PCR tests performed per population in Japan has been much lower compared with that in other countries and regions.^6,7^ This difference is because PCR testing was generally restricted to symptomatic patients and their close contacts, and testing was not made available to asymptomatic individuals without a medical need. However, PCR tests from private contractors have been available and widely used, but without obligation to report test results to the public health authorities, and therefore these cases have not necessarily been reflected in official figures. There has been speculation that the low case numbers in Japan may be due to the limited testing offered and reported in government figures. Moreover, early isolation to contain further infection should be initiated if or when an individual with an asymptomatic infection is identified, as persons with asymptomatic infection have been reported to disperse infectious amounts of virus (1 in 3 persons as reported for the UK).^8^

The extent of community infection can be ascertained by a population-based random sampling survey or sentinel surveillance in the general population, including asymptomatic individuals with SARS-CoV-2 infection.^9^ A random sampling survey is optimal to derive a population-representative estimate of SARS-CoV-2 infection, but costly and it is difficult to assess trends. However, a sentinel surveillance system can be used for screening purposes among high-risk groups and the general population, and population-representative case numbers may be derived from a weighted estimate of sentinel surveillance sites.^10,11^

The results from a large-scale sentinel screening program that was conducted in the general population in more than 1 million asymptomatic individuals in Japan are reported herein. The objectives of the program were 2-fold: to identify the trends and frequency of asymptomatic infections and assess the importance of PCR testing in the general population in Japan. With additional results of testing in symptomatic patients, the study aimed to provide evidence on the state of SARS-CoV-2 infection in the general population of Japan.

## Methods

### Sentinel Screening of Asymptomatic Individuals

All recruited individuals were asymptomatic and included children, students, employed adults, and older individuals, as well as volunteers for the testing program to broadly reflect the Japanese general population. Fourteen prefectures in Japan (Hokkaido, Miyagi, Tochigi, Saitama, Tokyo, Chiba, Kanagawa, Aichi, Gifu, Kyoto, Osaka, Hyogo, Fukuoka, and Okinawa prefectures) between February 22 and December 8, 2021, contributed. The third state of emergency was declared from April 25 to June 20, 2021, and the fourth state of emergency from July 12 to September 30, 2021. The Tokyo Olympics period was from July 23 to August 8, 2021.^12^ Sampling was conducted at offices, train stations, airports, downtown areas, business offices, factories, construction sites, kindergartens, schools, and student dormitories where there is potential increased risk of infection. Data on infected symptomatic persons and patients as diagnosed by PCR testing using nasopharyngeal swabs in medical institutions and health authorities were provided by the Ministry of Health, Labour, and Welfare of Japan.^13,14^ The infection rate by age group was calculated based on the 2020 census.^15^ eTable 2 and eFigure 1 in Supplement 1 were based on the Basic Resident Register of January 1, 2021. The present study conforms to principles of the Strengthening the Reporting of Observational Studies in Epidemiology (STROBE) reporting guideline for reporting observational studies. The program was implemented at the request of the Advisory Board of the COVID-19 Artificial Intelligence Simulation Project^16^ promoted by the Office for COVID-19 and Other Emerging Infectious Disease Control, Cabinet Secretariat, Government of Japan.^12^ Written consent was obtained when collecting samples and personal information. This investigation was not subject to ethical review as a matter of extraordinary public health (pandemic) under the guidance of the Ministry of Health, Labour, and Welfare of Japan. Data were anonymized and stored in the data repository of the data handling organizations (Mitsubishi Research Institute Inc and/or Dentsu Promotion Plus Inc).

### PCR Testing Method

Saliva samples were used for asymptomatic individuals (test kits provided by Healthcare Technologies Corp, Rakuten Group Inc, BML Inc, Kinoshita Group PCR Testing, J-VPD Inc, MBIC Life Co Ltd, Okinawa Private PCR Laboratory Co Ltd, and Mitaka Trade Co Ltd). Note that Japan uses saliva samples that show a high rate of agreement with nasopharyngeal swabs from patients with infection until the ninth day after onset of the infection.^17,18,19^ Samples were collected, stored at room temperature, and sent to the testing center on the day of collection; PCR tests were performed within 48 hours when possible. The criteria for a PCR test as being positive was a cycle threshold (Ct) value of approximately 40 as standard practice for Japan^20,21^ (range, 38-40; approximately 75% of tests used a Ct value of 40 and approximately 25% used a Ct value of 38). Note that the Ct value is vendor kit-specific and cannot be used for direct comparison as they differ according to kits but provide a general view of viral levels.^22^ Regarding the PCR testing using saliva samples, namely, on the specificity of the method, a study commissioned by the Japanese Ministry of Health, Labour, and Welfare has previously reported the validity of this approach.^23^ eTable 2 and eFigure 1 in Supplement 1 were based on the Basic Resident Register of the Ministry of Health, Labour and Welfare of January 1, 2021.

Demographic information was collected at the time of sample collection, and the individual was notified by email or by online portal of PCR results. Information on vaccination status was collected from the week of March 15, 2021.

### Statistical Analysis

Descriptive statistics were analyzed using Excel, version 2209 (Microsoft Corp). Summary data on infection rates have been published in part as a matter of public health during the pandemic on the website of the Office for COVID-19 and Other Emerging Infectious Disease Control, Cabinet Secretariat, Government of Japan.^12^

## Results

### Sample Characteristics

A total of 1 082 976 individuals were enrolled in the study. The sample included 52.08% males and 47.90% females (0.03% not reported). Demographic data, including the number of individuals, age, occupation, and region, are presented in eTable 1 in Supplement 1. The locations of the 14 prefectures (regions) where samples were collected are shown in eFigure 1 in Supplement 1. The population of these regions accounts for 62.6% of the national population (eTable 2 and eFigure 1 in Supplement 1), and 80.7% of patients with symptomatic infection nationwide with high correlation between the number of symptomatic patients in the 14 prefectures and nationwide (*R*^2^ = 0.998) (eFigure 2 in Supplement 1).

**Figure 1.**
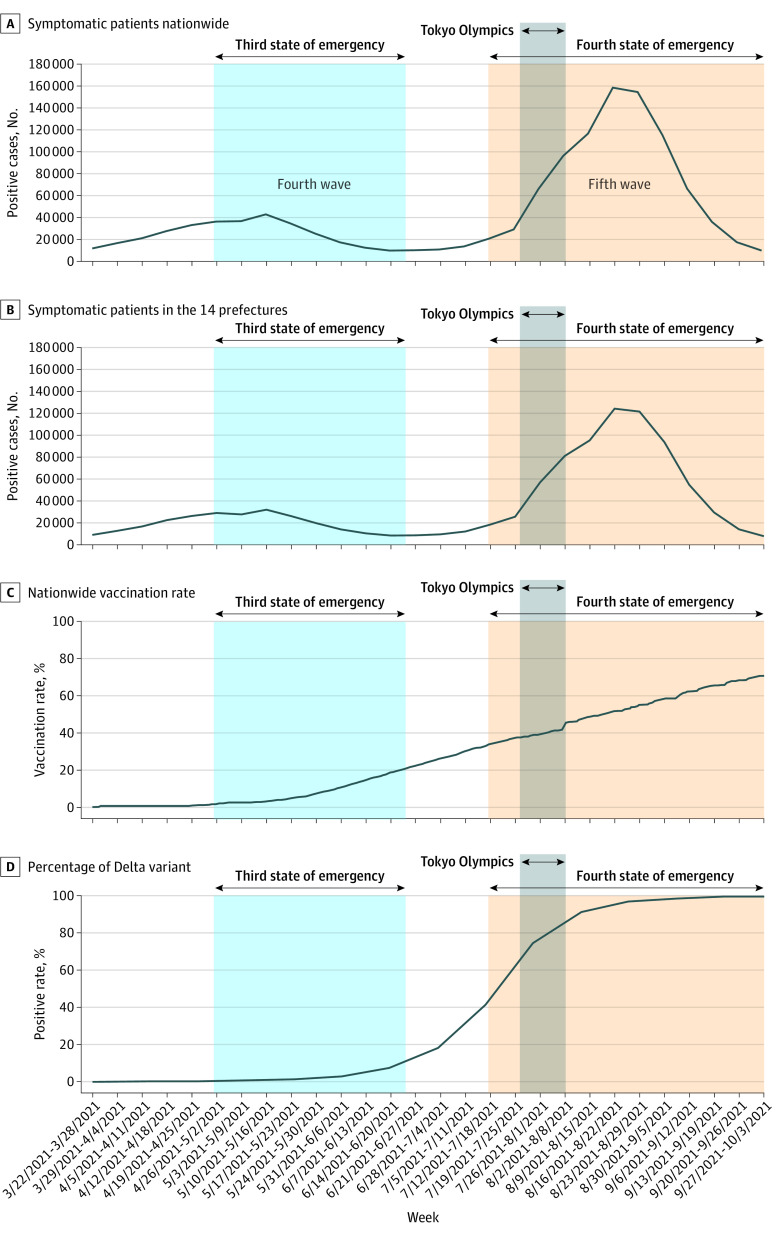
Baseline Serial Data During the Study Period on Infections, and Surge in Variant Strain and Vaccination Status Total reported number of symptomatic patients nationwide (A) and in the 14 prefectures (B), vaccination rate (nationwide) (C), and percentage of Delta variant (nationwide) (D).

Across age groups, there were fewer people aged 60 years and older (11.1% for the tested individuals vs 31.7% for the population of 14 prefectures) and those younger than 20 years (9.63% for the tested individuals vs 16.6%); therefore, the study population showed selection bias for these age groups (eTable 1 in Supplement 1). Baseline serial data during the study period on infections (both nationwide and in the 14 prefectures) and of vaccination status and surge in variant strain are shown in Figure 1. Figure 1A combines the data obtained by the sentinel surveillance for the 14 prefectures with national surveillance data, and Figure 1B presents only the sentinel surveillance data. The duration of this investigation overlaps the fourth (April to mid-June) and fifth (end of June to September) waves of infection in Japan in 2021, with the Tokyo Olympics being held during the latter period. During the fourth wave, the Alpha strain was predominant when vaccination had yet to be fully implemented. In contrast, the fifth wave coincided with the Delta strain outbreak with concurrent rapid rollout of vaccination.

### PCR Positive Rates According to Ct Value

**Figure 2.**
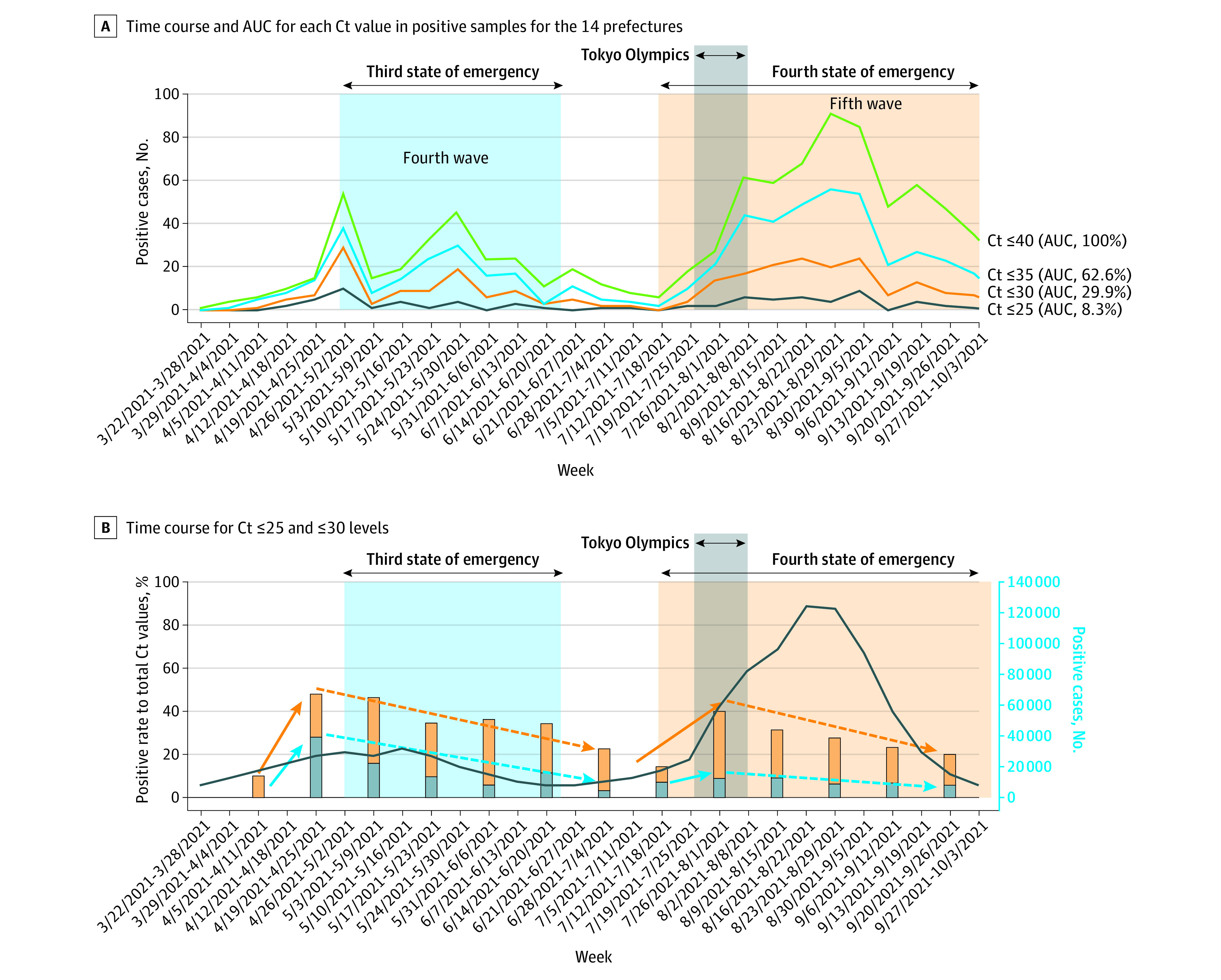
Time Course of Asymptomatic Infections According to Cycle Threshold (Ct) Value in 14 Prefectures A, Time course and the area under the curve (AUC) for each Ct value in positive samples for the 14 prefectures. B, Time course of percentage for Ct less than or equal to 25 (blue bars and dotted line) and Ct less than or equal to 30 levels (orange bars and dotted line) to total Ct values. The dark blue curve shows trends in the number of symptomatic infections in the 14 prefectures.

The PCR positive rate in tested asymptomatic individuals was approximately 0.03% (95% CI, 0.02%-0.05%) when the epidemic subsided, with a maximum of 0.33% (95% CI, 0.25%-0.43%) during the peak period of the fourth surge in May and the fifth surge in August 2021 (eTable 3 in Supplement 1), which suggests that the infection was more widespread than suggested by officially reported figures when taking into consideration asymptomatic individuals. The overall average prevalence throughout the investigation period was 0.1%. The criterion^20,24^ for PCR positive testing in Japan is a Ct value of approximately 40; however, if a Ct value of 25, as used elsewhere (eg, UK) is applied,^25,26,27^ the positive rate decreases to 0.03% during the peak period (Figure 2A). The proportion of individuals reporting a Ct value of 30 or less increased at the beginning of the fourth and fifth waves and preceded the peak of each surge (Figure 2B).

### Time Course Analysis According to Age Group and Vaccination Status

**Figure 3.**
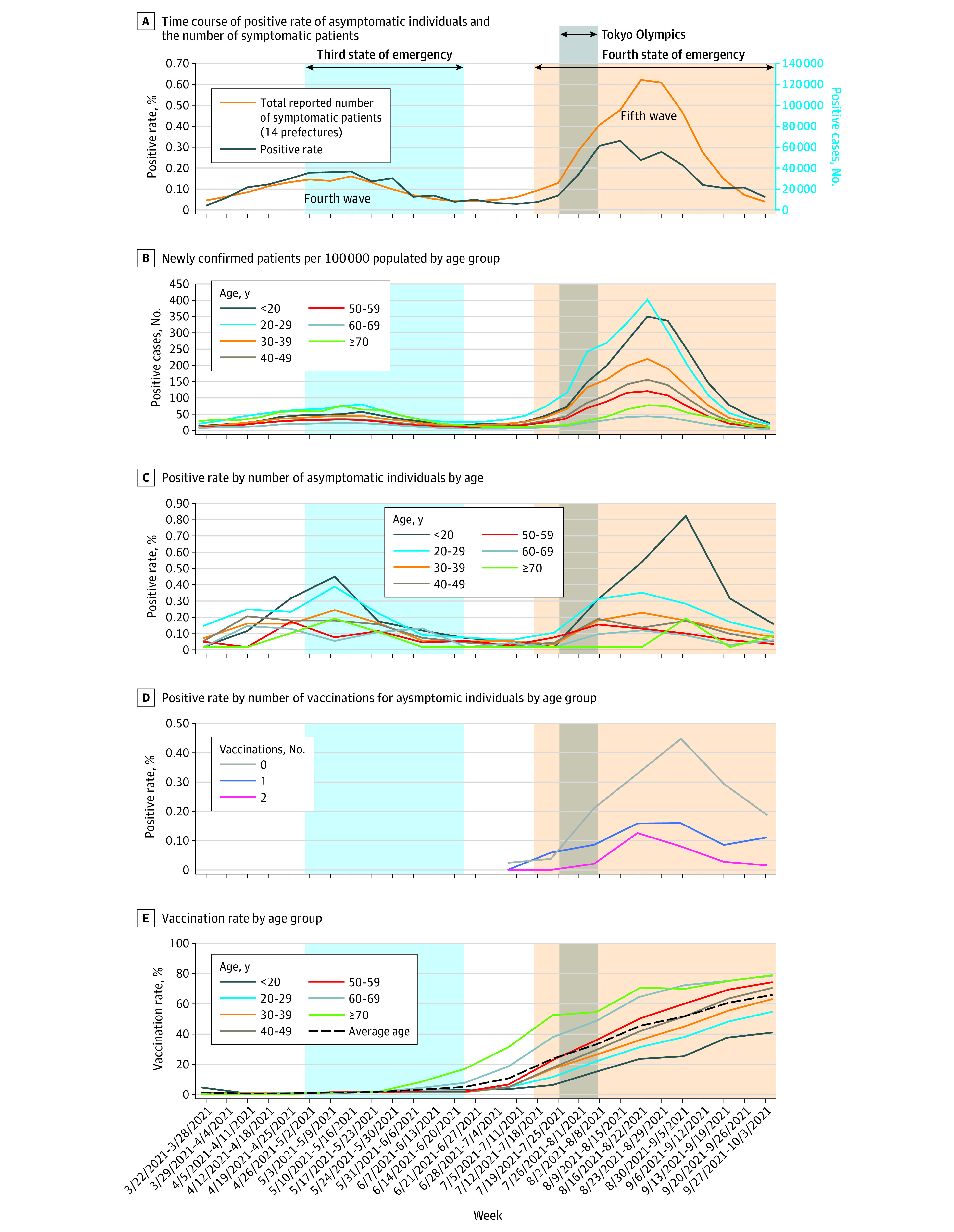
Time Course of Symptomatic Patients and Asymptomatic Individuals According to Age Group and Vaccination Status in the 14 Prefectures A, Time course of positive rate of asymptomatic individuals (blue) and the number of symptomatic patients (orange). B, Patients with newly confirmed infection per 100 000 census population by age group. C, Positive rate by number of asymptomatic individuals by age. D, Positive rate by number of vaccinations for asymptomatic individuals by age group. Note that the period before June 2021 was excluded due to insufficient data. E, Vaccination rate by age group.

Further analysis investigated the time course of symptomatic patients and asymptomatic individuals according to age group and vaccination status. There was a trend for an increased number of patients with infection in younger age groups during the fifth wave that coincided with an increased positive rate of individuals with asymptomatic infection in similar younger age groups (Figure 3A,B). The positive rate of asymptomatic individuals younger than 20 years and those in their 20s increased from April to the end of September, and the positive rate of those younger than 20 years was highest during the fifth wave (Figure 3C). The number of patients younger than 20 years was highest during the fifth wave in August (Figure 3B).

This also coincided with vaccination rollout, which showed an association of increased infection with lower vaccination status (more persons with 0 vaccination infected vs those once vaccinated once, followed by twice-vaccinated persons) (Figure 3D) and also noted more older individuals having higher vaccination rates (Figure 3E) as the national rollout continued. The national vaccination program progressed rapidly during the fifth wave, with a vaccination rate of 2.44% in the beginning of the week of May 22, which increased to 61.2% by the end of the week of October 4. The positive rate of vaccinated individuals with 2 doses was 0% to 0.12% and 0.05% to 0.2% for a single dose. The PCR positive rate in unvaccinated persons in August 2021 was 0.2% to 0.43%, which was higher than that of those who had already been vaccinated twice (Figure 3D). Collectively, an inverse association between vaccination rate and age group for asymptomatic infections and symptomatic patients was seen, with an association of younger age groups with lower vaccination rates showing more symptomatic patients and asymptomatic infections.

Japan's fourth wave was primarily due to the Alpha strain, while the Delta strain was predominant during the fifth wave (Figure 1A,D). The PCR positive rate of asymptomatic individuals and the number of infections in each region showed a high correlation during each surge (Figure 3A). However, during the fifth wave, the number of asymptomatic individuals with positive PCR results was higher than in the fourth wave. It is noteworthy that the association between asymptomatic testing and disease-based testing might vary with the virulence of the variant of concern and rollout of vaccination in the general population.

The fifth wave rapidly subsided after mid-August, which coincided with the summer holidays when the general flow of people in Japan decreased, but then mobility recovered to normal levels in about 2 weeks thereafter at the beginning of September. Increased immunity by the rapid vaccine rollout, along with seasonality and other viral characteristics, likely contributed to suppression of the epidemic rather than that of reduced human flow/mobility along with the effects of seasonality.

## Discussion

A population-based sentinel screening program of asymptomatic SARS-CoV-2 infection was conducted in over 1 million individuals in Japan to collect data on the state of SARS-CoV-2 infection in the population in Japan during 2021 (before the surge of the Omicron variant). The overall prevalence was generally low, varying from 0.33% during peak surges to 0.03% during the intermittent phase, with an overall average prevalence of 0.1% throughout the investigation period.

Polymerase chain reaction testing used a threshold criterion of a Ct value of approximately 40 as standard in Japan.^20,24^ However, if a Ct value of 25 were used, which is a decrease of at least 1000-fold for viral amplification as Japan uses stringent criteria for viral detection, the scale of infection would have been up to 10-fold less.

The state of SARS-CoV-2 infection in the community and population was more widespread than suggested by officially reported figures when taking into consideration asymptomatic individuals. Asymptomatic screening of the general population has been reported for university students (Boulder, Colorado) in 72 500 individuals using similar saliva sampling showing that 2% were positive on PCR testing with no reported symptoms.^28^ Viral load in asymptomatic individuals was reported to be indistinguishable from that in symptomatic patients with approximately one-half of asymptomatic individuals reporting a Ct value of 29, which would correspond to at least 100-fold more than what was observed in the present study.^28^ A nationwide study in the UK—the Real-Time Assessment of Community Transmission-1 study—done as a community-wide program that tracks the prevalence of COVID-19 cases across England through repeated random population-based sampling in approximately 600 000 cases between May 1 and September 8, 2020, when reported,^8^ examined volunteers regardless of symptoms and reported a prevalence of approximately 5% at peak periods (first wave in the UK), which decreased to 0.04% when lowest during the observation period. Studies in Asia had the lowest percentage at 0.05% (95% CI, 0.04%-0.07%),^9^ which may be the result of population-wide PCR screening programs as implemented in China. However, data on asymptomatic individuals with positive test results in the general population have varied, with a reported positive PCR rate of 0.003% (95% CI, 0.00270%-0.00339%) in 10 million residents of Wuhan,^29^ but others have reported up to 13.34% (95% CI, 10.86%-16.29%).^30^

Furthermore, a meta-analysis of asymptomatic infection in 29.8 million individuals from varied backgrounds and geographies suggested a positive rate of 0.2%, which is still at least 10-fold higher than in the present study (note that this meta-analysis included China, as Chinese-language publications were included).^9^ To our knowledge, therefore, the prevalence of SARS-CoV-2 infection in the general population in Japan based on the present study is one of the lowest reported to date.

The relatively low prevalence of COVID-19 cases in the Japanese population is likely due to a multitude of factors, including cultural and behavioral factors, in addition to potential biological factors. Japanese citizens have complied with voluntary protection measures, notably face masks, which are used both as protection from infection and to prevent further spread of the infection in a widespread public-health manner as a part of social culture and has most likely contributed to less spread of the virus.^31^ Combined rapid rollout of the vaccination program nationwide to 75.5% of the total population also likely contributed (note that this study predated current use of third booster vaccinations). It has also been reported that Japanese individuals may be more resilient to viral infection due to genetic background (eg, specific human leukocyte antigen type), which also might be attributed to biological protection.^32^ A state of emergency was declared in the described 14 prefectures that requested voluntary cooperation with social distancing and remote working in addition to shorter business hours for the retail and hospitality sectors, but a compulsory lockdown during the course of the pandemic has not been implemented in Japan to date.

An increase in asymptomatic individuals with positive test results with a Ct value of 25 or 30 or less preceding infection surges suggests that asymptomatic screening may be useful for identification of potential infection surges, at least when used in combination with a baseline Ct value of approximately 40 for surveillance. There was also value in identification of hotspots for infection in high-risk populations (eg, schools) where targeted asymptomatic testing in high-risk populations will be a potentially viable strategy moving forward.

### Limitations

This study has limitations. The sample population was generally reflective of the general population, but there were fewer people aged 60 years and over and those younger than 20 years, and therefore showed selection bias for these age groups (eTable 1 in Supplement 1). The vaccination rates and increasing trends among asymptomatic individuals who were tested were in agreement with the overall Japanese population (Figure 1C and Figure 3E); therefore, we believe that this sentinel surveillance sample selection was conducted in a balanced manner for population and regions.

## Conclusions

This cross-sectional study provides data on the prevalence of asymptomatic SARS-CoV-2 infection in the general population of Japan during the pandemic in 2021. A low rate of infection was seen in the Japanese population compared with reported levels elsewhere in the world.

This investigation has resulted in evidence generation for policy change by the Japanese government to implement PCR testing for asymptomatic individuals in high-risk populations in addition to symptomatic individuals by local authorities as needed and appropriate in current and future surges and future and emerging pandemics. This study also contributes to knowledge generation for improvement in situational awareness and will feed into the public health response even at low prevalence.

## Notes

Supplement 1. **eTable 1.** Demographic Characteristics **eTable 2.** Population and Proportion of the 14 Prefectures **eTable 3.** Positive Rate by Prefecture During Epidemic and Nonepidemic Periods **eFigure 1.** Location of the 14 Prefectures (A). Population Distribution by Prefecture (note that the Population of the 14 Prefectures Accounts for 62.63% of the National Population)(B) **eFigure 2.** Temporal Trends in the Number of Symptomatic Patients in the 14 Prefectures (blue) and Nationwide (orange)(A), and Correlation Analysis Between the Two (R2 = 0.998)(B)

Supplement 2.
Data Sharing Statement
