# Supplementary material for: Prevalence of Asymptomatic SARS-CoV-2 Infection in Japan
Source: JAMA Netw Open. 2022 Dec 27;5(12):e2247704. doi: 10.1001/jamanetworkopen.2022.47704 (PMC9856923; doi:10.1001/jamanetworkopen.2022.47704)
Supplement: Supplement 1. — eTable 1. Demographic Characteristics eTable 2. Population and Proportion of the 14 Prefectures eTable 3. Positive Rate by Prefecture During Epidemic and Nonepidemic Periods eFigure 1. Location of the 14 Prefectures (A). Population Distribution by Prefecture (note that the Population of the 14 Prefectures Accounts for 62.63% of the National Population)(B) eFigure 2. Temporal Trends in the Number of Symptomatic Patients in the 14 Prefectures (blue) and Nationwide (orange)(A), and Correlation Analysis Between the Two (R2 = 0.998)(B) [file jamanetwopen-e2247704-s001.pdf]

Supplementary Online Content

Suzuki T, Aizawa K, Shibuya K, et al. Prevalence of asymptomatic SARS-CoV-2 infection in Japan. *JAMA Netw Open*. 2022;5(12):e2247704.
doi:10.1001/jamanetworkopen.2022.47704

eTable 1. Demographic Characteristics

eTable 2. Population and Proportion of the 14 Prefectures

eTable 3. Positive Rate by Prefecture During Epidemic and Nonepidemic Periods

eFigure 1. Location of the 14 Prefectures (A). Population Distribution by Prefecture (note that the Population of the 14 Prefectures Accounts for 62.63% of the National Population)(B)

eFigure 2. Temporal Trends in the Number of Symptomatic Patients in the 14 Prefectures (blue) and Nationwide (orange)(A), and Correlation Analysis Between the Two (R^2 = 0.998)(B)

© 2022 Suzuki T et al. *JAMA Network Open.*

eTable 1. Demographic characteristics

	Number (%)		
Tested subjects	1,082,976 (100.00)		
Prefecture			
Hokkaido	62,624 (6.16)		
Miyagi	42,932 (4.22)		
Tochigi	37,163 (3.65)		
Saitama	76,644 (7.54)		
Tokyo	290,900 (28.61)		
Chiba	37,655 (3.70)		
Kanagawa	89,032 (8.76)		
Aichi	49,953 (4.91)		
Gifu	42,932 (4.22)		
Kyoto	41,842 (4.11)		
Osaka	104,411 (10.27)		
Hyogo	42,085 (4.14)		
Fukuoka	64,894 (6.38)		
Okinawa	53,582 (5.27)		
Age (range)	Tested subjects, n (%)	14 prefectures, n (%)	Nationwide, n (%)
0-19	81,828 (9.6)	12,701,264 (16.6)	21,037,894 (16.6)
20-29	208,984 (24.6)	8,301,115 (10.9)	12,822,884 (10.1)
30-39	134,158 (15.8)	9,162,513 (12.0)	14,374,823 (11.3)
40-49	169,923 (20.0)	11,570,585 (15.1)	18,425,929 (14.5)
50-59	160,291 (18.9)	10,425,914 (13.6)	16,811,709 (13.3)
60-69	75,920 (8.9)	8,744,859 (11.4)	15,510,948 (12.2)
>70	18,779 (2.2)	15,539,547 (20.3)	27,669,999 (21.8)
Gender			
Male	446,074 (52.08)		
Female	410,240 (47.90)		
NA	220 (0.03)		
Social background/setting			
Student (including children)			
Pre-school	106,715 (9.85)		
Primary	10,921 (1.01)		
Secondary	5,678 (0.52)		
University	152,216 (14.06)		
Worker			
Office-based	286,340 (26.44)		
Non-office-based	76,629 (7.08)		
Other	172,484 (15.93)		

© 2022 Suzuki T et al. *JAMA Network Open.*

eTable 2. Population and proportion of the 14 prefectures

	Population	Ratio (weight) to the total population of Japan (%)	Ratio (weight) to 14 prefectures (%)
Nationwide	126,654,244	100	
Prefecture			
Hokkaido	5,228,732	4.13	6.59
Miyagi	2,282,106	1.80	2.88
Tochigi	1,955,402	1.54	2.47
Saitama	7,393,849	5.84	9.32
Tokyo	6,322,897	4.99	7.97
Chiba	13,843,525	10.93	17.45
Kanagawa	9,220,245	7.28	11.62
Aichi	2,016,868	1.59	2.54
Gifu	7,558,872	5.97	9.53
Kyoto	2,530,609	2.00	3.19
Osaka	8,839,532	6.98	11.14
Hyogo	5,523,627	4.36	6.96
Fukuoka	5,124,259	4.05	6.46
Okinawa	1,485,484	1.17	1.87
14 prefectures	79,326,007	62.63	100
Others	47,328,237	37.37	

© 2022 Suzuki T et al. *JAMA Network Open.*

eTable 3. Positive rate by prefecture during epidemic and nonepidemic periods

	**Prevalence**	
	**Lowest positive rate (2021/7/5-7/11)**	**Highest positive rate (2021/8/9-8/15)**
	**Positive rate (95% CI), %**	**Positive rate (95% CI), %**
Prefecture		
Hokkaido	0.00 (0.00–0.11)	0.34 (0.13–0.87)
Miyagi	0.06 (0.01–0.34)	0.00 (0.00–1.62)
Tochigi	0.00 (0.0–0.66)	0.00 (0.00–3.47)
Saitama	0.00 (0.0–0.19)	0.00 (0.00–0.30)
Tokyo	0.06 (0.03–0.14)	0.29 (0.19–0.45)
Chiba	0.00 (0.00–0.35)	2.62 (1.64–4.15)
Kanagawa	0.04 (0.01–0.23)	0.19 (0.06–0.56)
Aichi	0.08 (0.01–0.44)	0.18 (0.03–1.03)
Gifu	0.06 (0.01–0.37)	0.00 (0.00–1.02)
Kyoto	0.00 (0.00–0.29)	0.29 (0.08–1.05)
Osaka	0.00 (0.0–0.18)	0.20 (0.07–0.60)
Hyogo	0.00 (0.0–0.14)	0.21 (0.04–1.17)
Fukuoka	0.00 (0.0–0.14)	0.62 (0.24–1.58)
Okinawa	0.00 (0.0–3.23)	0.16 (0.04–0.58)
14 prefectures	0.03 (0.02–0.05)	0.33 (0.25–0.43)

© 2022 Suzuki T et al. *JAMA Network Open.*

eFigure 1. Location of the 14 prefectures (A). Population distribution by prefecture (note that the population of the 14 prefectures accounts for 62.63% of the national population)(B).

A

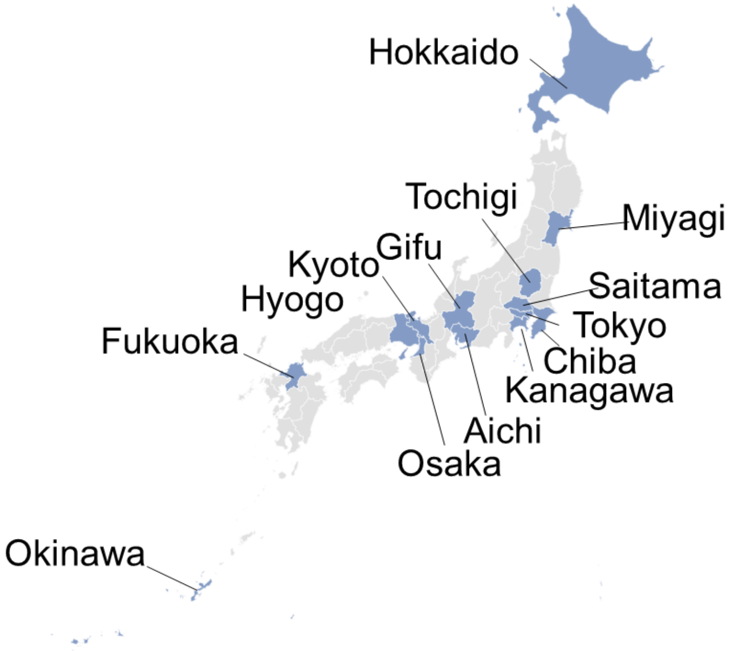

B

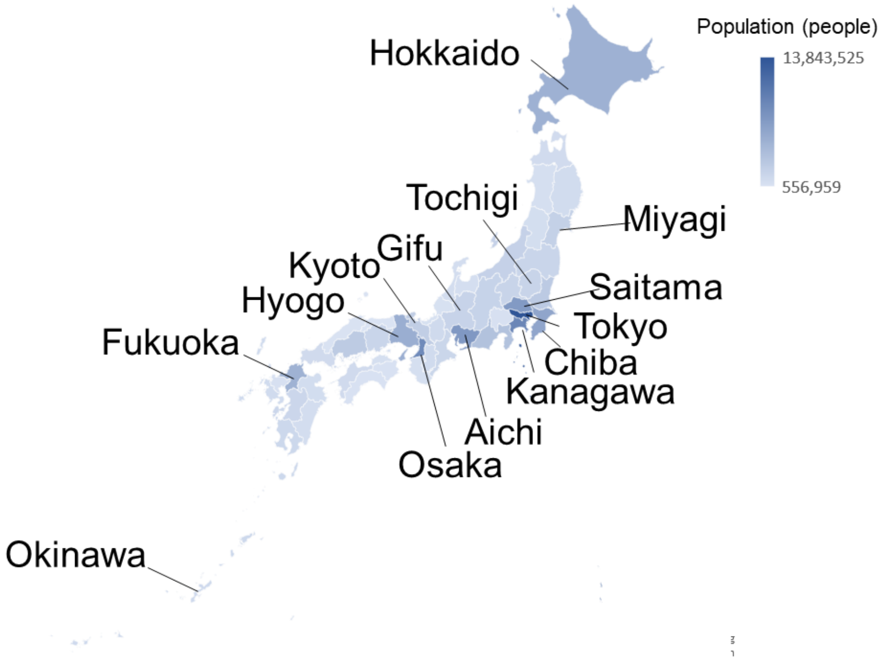

© 2022 Suzuki T et al. *JAMA Network Open.*

eFigure 2. Temporal trends in the number of symptomatic patients in the 14 prefectures (blue) and nationwide (orange)(A), and correlation analysis between the two (R^2=0.998)(B).

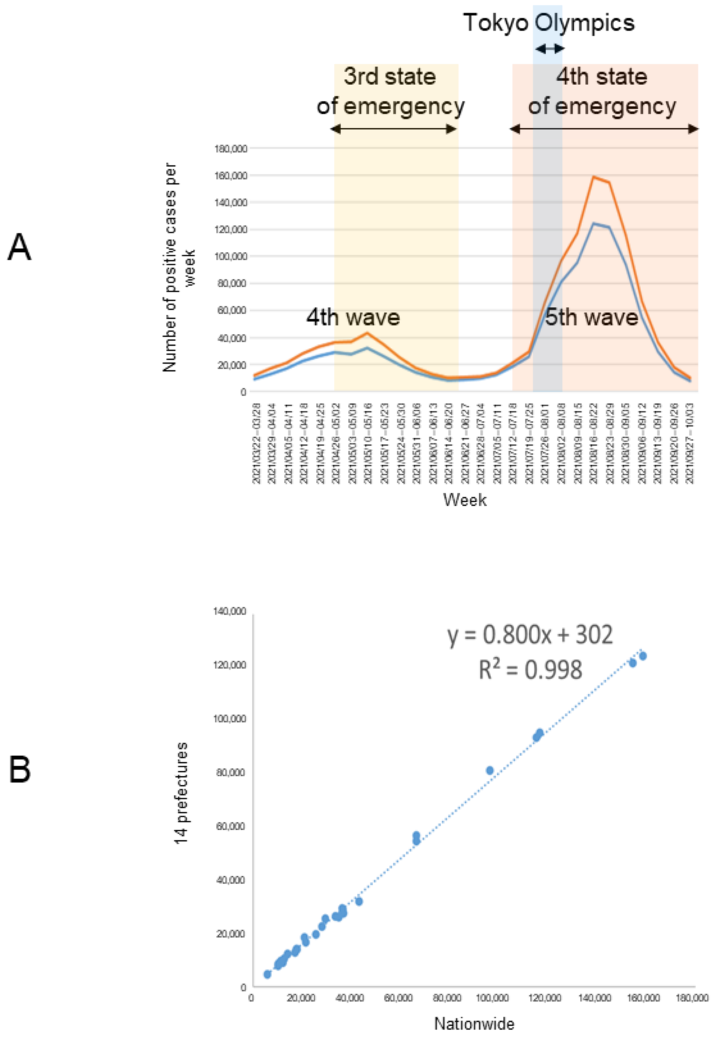

© 2022 Suzuki T et al. *JAMA Network Open.*
