# Supplementary material for: Prevalence of Asymptomatic SARS-CoV-2 Infection in Japan
Source: JAMA Netw Open. 2022 Dec 27;5(12):e2247704. doi: 10.1001/jamanetworkopen.2022.47704 (PMC9856923; doi:10.1001/jamanetworkopen.2022.47704)
Supplement: Supplement 2. — Data Sharing Statement [file jamanetwopen-e2247704-s002.pdf]

Data Sharing Statement

Suzuki. Prevalence of Asymptomatic SARS-CoV-2 Infection in Japan. *JAMA Netw Open*. Published December 27, 2022. doi:10.1001/jamanetworkopen.2022.47704

Data
Data available: No
